# MicroRNA-145 inhibits migration and invasion via inhibition of fascin 1 protein expression in non-small-cell lung cancer cells

**DOI:** 10.3892/mmr.2021.12365

**Published:** 2021-08-12

**Authors:** Yingying Zhang, Xucheng Yang, Haijun Wu, Weibin Zhou, Zhenzhen Liu

Mol Med Rep 12: 6193-6198, 2015; DOI: 10.3892/mmr.2015.4163

Following the publication of this paper, it was drawn to the Editors’ attention by a concerned reader that certain of the Transwell cell migration data shown in Fig. 4 were strikingly similar to data appearing in different form in other articles by different authors. Owing to the fact that the contentious data in the above article had already been published elsewhere, or were already under consideration for publication, prior to its submission to *Molecular Medicine Reports*, the Editor has decided that this paper should be retracted from the Journal. The authors were asked for an explanation to account for these concerns, but the Editorial Office did not receive any reply. The Editor apologizes to the readership for any inconvenience caused.

